# Fast, Easy, and Reproducible Fingerprint Methods for Endotoxin Characterization in Nanocellulose and Alginate-Based Hydrogel Scaffolds

**DOI:** 10.1021/acs.biomac.4c00989

**Published:** 2024-09-12

**Authors:** Jan Zuber, Paula Lopes Cascabulho, Sara Gemini Piperni, Ronaldo José Farias Corrêa do Amaral, Carla Vogt, Vincent Carre, Jasmine Hertzog, Eero Kontturi, Anna Trubetskaya

**Affiliations:** † Department of Analytical Chemistry, TU Freiberg, Leipziger Street 29, 09599 Freiberg, Germany; ‡ Faculty of Medicine, Federal University of Rio de Janeiro, Avenida Carlos Chagas Filho 373, 21941-853 Rio de Janeiro, Brazil; § Laboratory of Cellular Proliferation and Differentiation, Institute of Biomedical Sciences, Federal University of Rio de Janeiro, Avenida Carlos Chagas Filho 373, 21941 Rio de Janeiro, Brazil; ∥ Laboratory of Biomineralization, Institute of Biomedical Sciences, Federal University of Rio de Janeiro, Avenida Carlos Chagas Filho 373, 21941 Rio de Janeiro, Brazil; ⊥ Laboratory of Biotechnology, Bioengineering and Nanostructured Biomaterials, Institute of Biomedical Sciences, Federal University of Rio de Janeiro, Avenida Carlos Chagas Filho 373, 21941 Rio de Janeiro, Brazil; # Université de Lorraine, LCP-A2MC, 1 Boulevard Arago, 57000 Metz, France; ∇ Department of Bioproducts and Biosystems, Aalto University, Vuorimiehentie 1, 02150 Espoo, Finland; ○ Department of Biosciences, Nord University, Kongensgate 42, 7713 Steinkjer, Norway; ◆ Department of Engineering, University of Limerick, Castletroy, Co. Limerick V94T9PX, Ireland

## Abstract

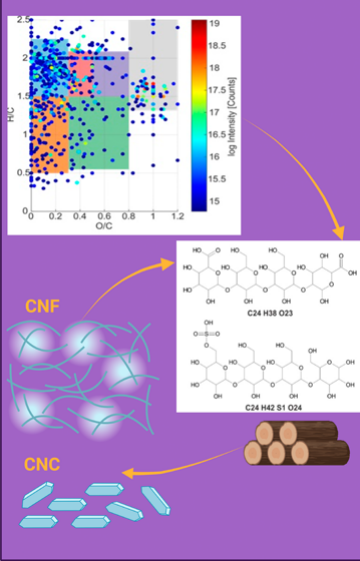

Nanocellulose- and alginate-based hydrogels have been suggested as potential wound-healing materials, but their utilization is limited by the Food and Drug Administration (FDA) requirements regarding endotoxin levels. Cytotoxicity and the presence of endotoxin were assessed after gel sterilization using an autoclave and UV treatment. A new fingerprinting method was developed to characterize the compounds detected in cellulose nanocrystal (CNC)- and cellulose-nanofiber (CNF)-based hydrogels using both positive- and negative-ion mode electrospray ionization Fourier transform ion cyclotron resonance mass spectroscopy (ESI FT-ICR MS). These biobased hydrogels were used as scaffolds for the cultivation and growth of human dermal fibroblasts to test their biocompatibility. A resazurin-based assay was preferred over all other biocompatibility methodologies since it allowed for the evaluation of viability over time in the same sample without causing cell lysis. The CNF dispersion of 6 EU mL^–1^ was slightly above the limits, and it did not affect the cell viability, whereas CNC hydrogels induced a reduction of metabolic activity by the fibroblasts. The chemical structure of the detected endotoxins did not contain phosphates, but it encompassed hydrophobic sulfonate groups, requiring the development of new high-pressure sterilization methods for the use of cellulose hydrogels in medicine.

## 1 Introduction

Nanocellulose, methylcellulose, and alginate are frequently selected as the main compounds in three-dimensional gels for biomedical applications. However, the presence of endotoxins, known as bacterial lipopolysaccharides (LPS), in these compounds may hamper their therapeutic applications. For instance, LPS (commonly present in both naturally derived and synthetic biomaterials) is harmful to successful wound healing by potentially inducing an inflammatory response and a hypertrophic scar. The hydrogels for wound-healing applications hold paramount importance in addressing the challenges associated with conventional wound care strategies, such as infection control, moisture management, lack of tailored/customized therapy for specific wounds, and scar formation.^1^ Biobased hydrogels have garnered particular attention in the field due to their biodegradability and biocompatibility.^2^ They offer a versatile platform for the development of advanced wound dressings and tissue engineering scaffolds.^3^ The high water content, tunable mechanical properties, and ability to promote cell adhesion and proliferation make them promising candidates for promoting wound-healing processes.^4^ However, many metals and biopolymers demonstrated a high affinity for endotoxins as their cationic nature predisposes its interaction with the negatively charged phosphate groups in LPS.^5^

Endotoxins are heat-stable molecules (<180 °C) that can be found in the outer membrane of Gram-negative bacteria and contain lipid A, core oligosaccharides, and an O-antigen.^6^ Endotoxins are major components of the outer leaflet of the outer membrane of Gram-negative bacteria and are composed of fatty acids, carbohydrates, phosphates, and a broad range of associated metal ions. Most endotoxins are related to LPS, while delta-endotoxin proteins, which are produced by *Bacillus thuringiensis*, are pore-forming toxins and have an exceptionally different structure from LPS.

Endotoxins have an anionic structure due to their highly phosphorylated inner core. Innate immune cells recognize the lipid A portion of endotoxin through toll-like receptor-4 (TLR4), which initiates downstream signaling followed by the release of proinflammatory cytokines and subsequent activation of immune responses.^7^ The host immune system can detect small concentrations of endotoxins, e.g., monocytes and dendritic cells (0.05–0.1 EU/mL).^8^ The Food and Drug Administration (FDA) guidelines indicate endotoxin limits of 0.5 EU mL^–1^, which are strictly followed in the medical sector.^9^

Nanocellulose has a unique nanoscale structure that mainly includes cellulose nanocrystals (CNCs), and cellulose nanofibrils (CNFs), which can be prepared by mineral acid hydrolysis and mechanical defibrillation (microfluidization). The cytotoxicity of nanocellulose-based materials was debatable in previous studies.^10^ The feedstock type, preparation method, and chemical modifications of nanocellulose determine the physical–chemical properties of the final product, thereby affecting the cytotoxic behavior of biobased hydrogels to biological cells.^11^ Leachable (1,3)-β-d-glucans are another important group of impurities in nanocellulose-based materials that can cause strong immune responses on biobased hydrogels in medicine.^12^

The endotoxin concentrations have traditionally been identified using biosensors, Limulus amebocyte lysate (LAL), or rabbit pyrogen tests.^13^ The chemical analysis of endotoxins is biased due to the strong dependency on human factors, which requires the development of new spectroscopic methods. Previous attempts have been made to identify structural differences between endotoxins in water using Py-GC/MS.^14^ Several chemical methods involving GC/MS techniques have been studied and developed for the structural identification of endotoxins.^15^ However, the concentrations of endotoxins determined with GC/MS methods are often higher than those obtained with LAL tests. In addition, GC/MS requires a long preparation time due to the need for derivatization of the sample.

HPLC-MS/MS is recognized as suitable for the analysis of aqueous samples, does not require derivatization, and does not expose the analytes to high temperatures, thus avoiding the risk of degradation. However, this method does not improve the limits of detection and quantification of LPS.^16^ The ionization of nanocellulose creates additional challenges for the identification of endotoxins in CNF and CNC solutions using spectroscopic methods.

In this study, a molecular fingerprint method using electrospray ionization Fourier transform ion cyclotron resonance mass spectroscopy (ESI FT-ICR MS) was applied to characterize endotoxins in nanocellulose, alginate, and methylcellulose-based hydrogels. This method provides the highest mass resolving power (up to 10^6^) and mass accuracy (<1 ppm) of any mass analyzer in the range of 100–10,000 *m*/*z*. Previous reports on the detection of polyaromatic and aliphatic compounds using ESI-MS have established ESI-MS as an effective approach for the characterization of byproducts from thermochemical processes in the range of 250–550 °C.^17−19^ The purpose of this work is to characterize the CNC and CNF-based hydrogel samples by ESI FT-ICR MS to highlight the chemical diversity of their LPS content. Knowledge of the chemical nature of endotoxins in biobased materials is critically important to tackle the issues associated with the development of processes to remove endotoxins from medical devices. This is because sterilization methods can frequently change the hydrogel scaffold properties or only partially remove the endotoxins.

## 2 Materials and Methods

### 2.1 Materials

Cellulose nanocrystals (CNCs) (*L*: 190 ± 50 nm; *d*: 7 ± 5 nm) obtained from hydrochloric acid hydrolysis of birchwood, cellulose nanocrystals (*L*: 103 ± 63 nm; *d*: 15 ± 5 nm) from sulfuric acid hydrolysis of Whatman paper filters (catalog number WHA1001125, 125 mm diameter) and cellulose nanofibers (CNFs) (*L*: 0.85 ± 0.35 μm; *d*: 3.5 ± 0.5 nm) from TEMPO-mediated oxidation were supplied by VTT (Finland).^20^ Polyethylene glycol (PEG, 35 kDa), polyethylenimine (PEI, 600 kDa), alginic acid (Sigma-Aldrich, 71238, 100–200 kDa), calcium chloride (CaCl_2_) dehydrate (Sigma-Aldrich, 223506), and Triton X-100 surfactant were supplied by Sigma-Aldrich (Finland). MC156S (DS = 1.4; 68 kDa) and SGA150 (DS = 1.86; 175 kDa) methylcellulose were donated by the US brand of Dow Chemicals.

Dulbecco’s modified Eagle’s medium (DMEM), sodium chloride (NaCl, 5 M), 4-(2-hydroxyethyl)-1-piperazineethanesulfonic acid (HEPES, 1 M), penicillin–streptomycin (10,000 U mL^–1^), Gibco TrypLE Select enzyme, Gibco GlutaMax, and Gibco fetal bovine serum (FBS) were supplied by Thermo Fisher Scientific (U.K.). Human dermal fibroblasts were obtained from the skin of healthy donors.

### 2.2 CNC and CNF Preparation

CNC dispersion (1 wt %, 1 mmol g^–1^) containing 1.0 mmol of −COOH per gram cellulose was obtained from HCl gas-hydrolyzed birchwood pulp followed by TEMPO-mediated oxidation, as reported previously.^21^ CNC dispersion (1 wt %, 0.064 mmol g^–1^) was obtained from 64% H_2_SO_4_ hydrolyzed Whatman paper filter (45 °C, 30 min reaction time, acid-to-cellulose ratio of 8.75–17.5). CNF (1 wt %) suspension was obtained from the alkaline oxidation of softwood with hypochlorite, catalyzed by TEMPO.^20^ Then, TEMPO-oxidized pulp was fibrillated using a high-pressure fluidizer Microfluidics M-110EH-30 (Microfluidics Inc.), equipped with two Z-type chambers (400 and 100 μm). The pulp was passed through the fluidizer two times at 1850 bar. The charge of the oxidized pulp, as measured by a standard conductometric titration procedure (SCAN-CM 65:02, 2002), was 0.836 mmol g^–1^.^22^

### 2.3 Hydrogel Preparation

**Figure 1:**
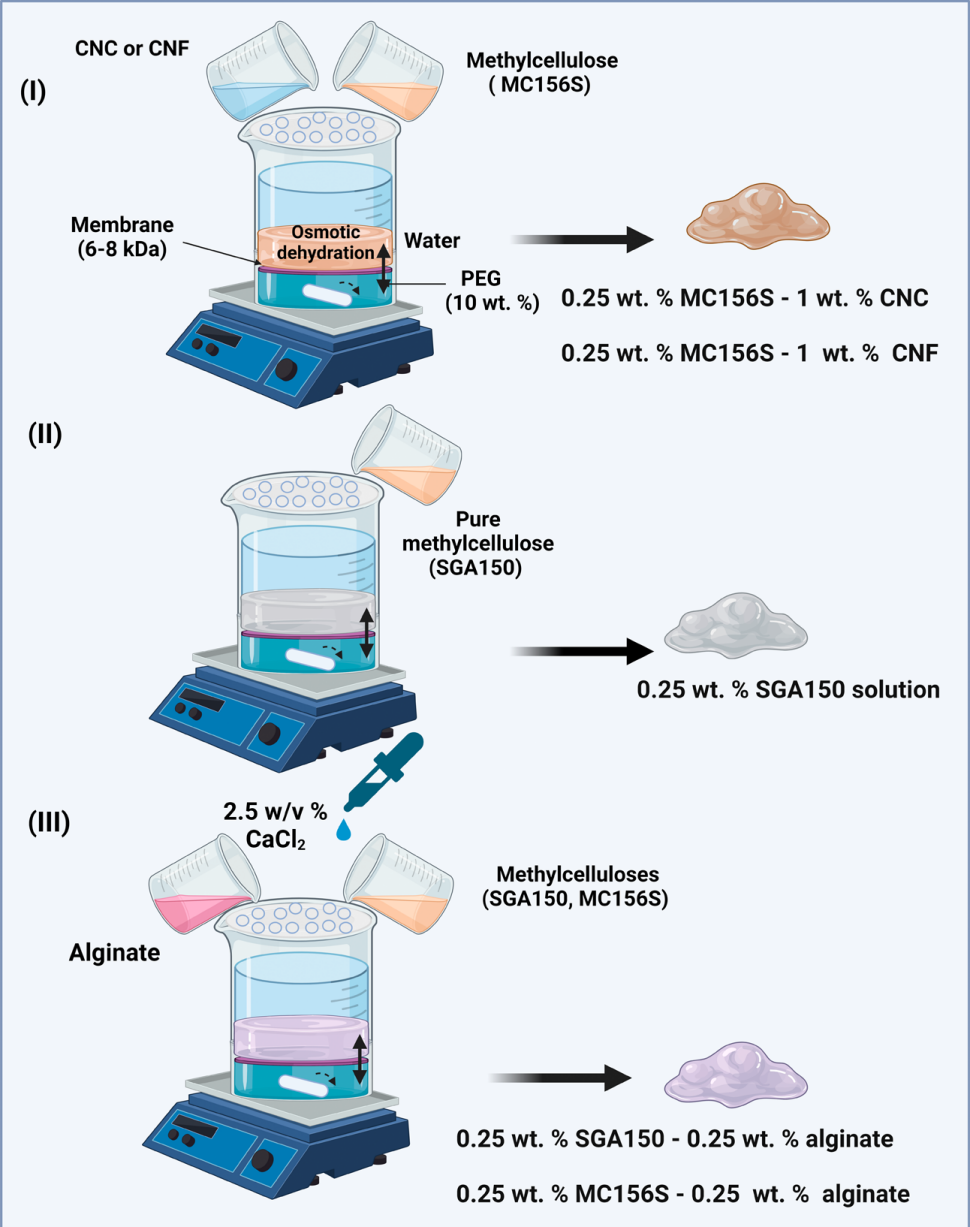
Preparation of hydrogels by the osmotic dehydration process with the following compounds: (A) 0.25 wt % methylcelluloses (MC156S, SGA150) and CNC/CNF; (B) pure methylcellulose SGA150; and (C) methylcellulose (MC156S; SGA150) and alginate with CaCl_2_ as a cross-linker.

Hydrogels were prepared at the laboratory scale using the osmotic dehydration principle.^23^ The setup consisted of two disposable plastic cups. The upper cup was covered with paraffin with multiple holes to ensure reproducible dehydration for 24 h. PEG solution was prepared by simply mixing a proper amount of solid PEG with water to obtain hydrogels with the desired mechanical properties to regulate the cell behavior. The solution was then mixed with a magnetic stirrer until the complete dissolution of PEG. The bottom of one cup was cut off and replaced by a dialysis membrane (Spectrum Laboratories Spectra/Por 1, U.K., cut off: 6–8 kDa). 15.6 mL aliquot of 10 wt % PEG solution was used as the water absorbent and added to the other cup with a magnetic stir bar inside. Then, a cup with the attached dialysis membrane was added to the PEG solution surface. A mixture of 0.25 wt % methylcellulose (MC156S; SGA150), CNF, TEMPO-oxidized CNCs, or alginate with several drops of 2.5 w/v % CaCl_2_ forming a total volume of 15.6 mL was added to the cup with an attached osmotic membrane, followed by placing the whole setup on a magnetic plate with constant stirring at room temperature (see Figure 1). Table 1 lists the hydrogels used in the experiments.

**Table 1:** Hydrogels Used in This Study

sample abbreviation	methylcellulose	alginate	CNC	CNF	CaCl_2_
A	0.25 wt % MC156S	0.25 wt %			2.5 w/v
B	0.25 wt % MC156S			1 wt %	
C	0.25 wt % SGA150				
D	0.25 wt % SGA150	0.25 wt %			2.5 w/v
E	0.25 wt % MC156S		1 wt %		

In the biocompatibility studies, a TEMPO-oxidized carboxylated CNC sample was used.

The solid content of the hydrogels was measured by drying the samples immediately after dehydration in an oven at 105 °C for 24 h. The weight was recorded before and after with a balance of five significant digits. Two independent measurements were performed. The hydrogels were sterilized at 121 °C for 20 min in an autoclave, and prior to each biocompatibility experiment, UV treatment was performed for 40 min on all hydrogels. All glass dishes and small laboratory instruments were sterilized at 200 °C for 60 min before the biocompatibility tests.

### 2.4 Determination of Endotoxins in Nanocellulose

The endotoxin content was verified using a ToxinSensor Chromogenic LAL Endotoxin Assay Kit (Genscript, Piscataway, NJ). After the test, the endotoxin concentrations were calculated according to the manufacturer’s guidelines using the formula 0.2618 × *x* – 0.0012 (where *x* is the absorbance at 545 nm of each cellulose sample). Commercially available endotoxin standards (lipopolysaccharide, LPS; 0.5 EU/mL) and LAL water were used as positive and negative controls, respectively.

### 2.5 Cell Culture and Biocompatibility Studies

Human dermal fibroblasts were obtained from the skin of healthy donors as a surgical waste of plastic surgery following informed consent and according to the ethics committee of the Associação Fluminense de Educação, Rio de Janeiro, Brazil, n°5.615.152. Cells were isolated as previously described.^24^

The human dermal fibroblasts obtained were cultured at 37 °C in a humidified atmosphere of 5% CO_2_ in Dulbecco’s modified Eagle’s medium with low glucose (DMEM, Sigma-Aldrich, Brazil) supplemented with 10% fetal bovine serum (Cultilab, Brazil). Indirect and direct cell viability assays were performed on cells at passage 4, with fibroblasts seeded on a 96-well plate at a density of 1 × 10^3^/well. In the indirect assay,^25^ 100 μL of each hydrogel was placed in the wells of 96-well plates and allowed to cross-link at 37 °C. After jellification, 100 μL of the cell culture medium was added to each sample and incubated at 37 °C for 24 h. This extract/conditioned medium was collected and transferred to a fibroblast culture to analyze its effect on cell viability (*n* = 4 technical replicates per condition from one independent experiment). In this scenario, a reduction in cell viability of more than 30% was considered cytotoxic. For the direct assay, 100 μL of each hydrogel was placed in each well of a 96-well plate and allowed to cross-link at 37 °C. After jellification, 100 μL of the cell culture medium containing fibroblasts at a density of 1 × 10^3^/well was added to each well (*n* = 8 technical replicates per condition from two independent experiments). For both indirect and direct assays, cell viability was measured with a Resazurin-based in vitro toxicology assay kit (TOX8, Sigma-Aldrich, Brazil) after 24 and 48 h of culture according to the manufacturer’s instructions. The absorbance values of all samples were normalized to control values at 48 h, which was considered viable. Values are expressed as mean ± standard error of the mean. Statistical significance was considered when *p* < 0.05 after a two-way ANOVA with multiple comparison tests. Finally, after 48 h of cell culture on gels in the direct assay, contrast phase microscopy images were obtained using a CKX41 microscope (Olympus) equipped with an EP50 digital camera to evaluate cell morphology.

### 2.6 Preparation of Nanocellulose for ESI-MS Analyses

For the mass spectrometric analyses, digestion solutions of TEMPO-oxidized carboxylated CNC and sulfated CNC solutions were first diluted 10-fold using methanol (Merck Supelco, LiChrososolv, purity ≥99.8%). These solutions were then filtered using a PTFE syringe filter (VWR, pore size of 0.22 μm) and diluted, once again, 10-fold with methanol, which finally led to a sample concentration of 1/100 in methanol that was used for the MS investigations. The TEMPO-oxidized CNF was prepared by weighing 10 mg in an amber glass vial. Afterward, 1 mL of methanol was added to the CNF sample, and the mixture was sonicated for 2 min in an ultrasonic bath. This sample mixture was also filtered through a PTFE syringe filter (VWR, pore size 0.22 μm), and then the filtrate was directly used for characterization by ESI FT-ICR MS.

### 2.7 FT-ICR-MS Analyses and Data Processing

All methanol solutions of the two CNC, one CNF sample, and a blank sample (pure methanol) were analyzed using a 15 T solariX FT-ICR-MS (Bruker Daltonics), equipped with an ESI source. The ESI source was operated in positive- and negative-ion mode, using the following parameters: capillary voltage, +2800 V (ESI(−))/–3000 V (ESI(+)); end plate offset, −500 V; nebulizer pressure, 1.0 bar; dry gas flow, 4.0 L/min; dry gas temperature, 300 °C; and syringe flow, 10 μL/min. Mass spectra were acquired in the *m*/*z* range of 153.48–2000.00 by accumulating 128 scans. The resulting data sets had a size of 8 M, and the resolving power was *R* = 800,000 at *m*/*z* = 400.

Peak picking, calibration, and molecular formula assignment were conducted using Bruker Daltonics software DataAnalysis 5.0 (SR 1). The mass calibration of the ESI FT-ICR MS experiments was conducted via a two-step process. In the first step, existing calibration lists were used for the first internal calibration. Molecular formulas were calculated from these mass spectra, and the resulting molecular formula lists were used to create an internal calibration list that contained molecular ions typical for the analyzed CNC and CNF samples. Molecular formulas were assigned to peaks with a signal-to-noise ratio (*s*/*n*) ≥ 10. The deviation from the theoretical masses should not exceed 0.3 ppm, and the molecular formulas should show a predefined elemental composition (C_c_H_h_N_n_O_o_S_s_Na_na_K_k_Cl_Cl_: *c* = unlimited, *h* = unlimited, 0 ≤ *n* ≤ 3, *o* = unlimited, 0 ≤ *s* ≤ 5, 0 ≤ na ≤ 10, 0 ≤ *k* ≤ 10, 0 ≤ cl ≤ 10). The exported peak and molecular formula lists were imported into MATLAB R2024a (Mathworks), further processed, and visualized using in-house scripts for blank correction and molecular formula filtering. Filtering of The molecular formula lists were filtered applying the rules established previously (double bond equivalent (DBE) ≥ 0, 0.3 ≤ H/C ≤ 2.5, O/C ≤ 1.2, N/C ≤ 1.0, S/C ≤ 1.0).^26,27^

## 3 Results

### 3.1 Biocompatibility of Hydrogels

In the indirect assay, no hydrogel treatment led to a reduction in cell viability of more than 30%; hence, no sample could be considered cytotoxic after 24 or 48 h, as shown in Figure 1.

**Figure 2:**
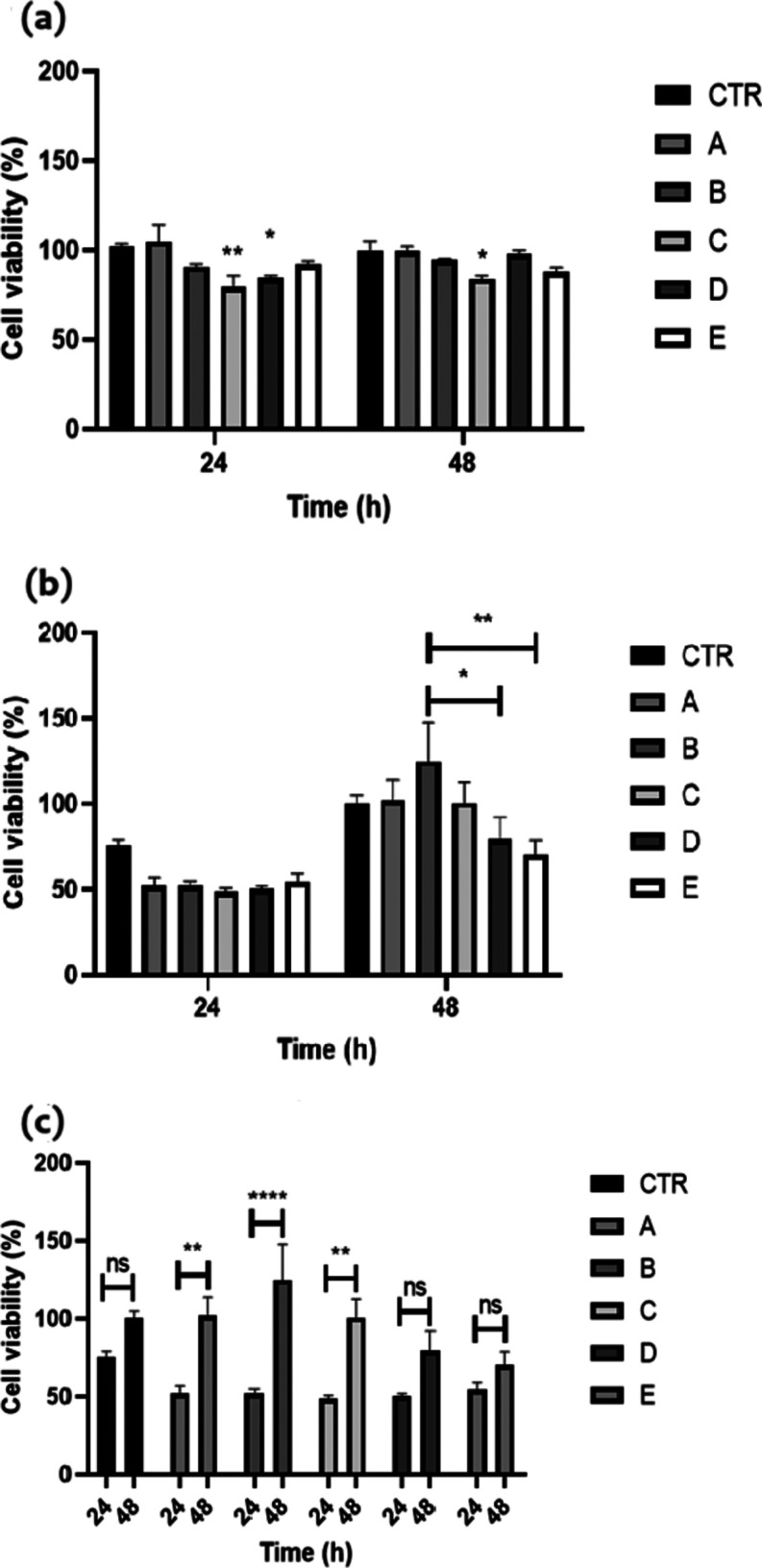
Effects of cellulose hydrogels on human dermal fibroblast viability. (a) Indirect assay, in which fibroblasts are treated in a conditioned medium and incubated for 24 h in the presence of the hydrogels. (b, c) Direct assay, in which fibroblasts are cultured directly on the hydrogels: (b) with testing of all hydrogels at 24 and/or 48 h. (c) Visualization of cell viability of each hydrogel at 24 and 48 h. The site letters mean (CTR) control medium; (A) 0.25 wt % MC156S–0.25 wt % alginate; (B) 0.25 wt % MC156S–1 wt % CNF; (C) 0.25 wt % SGA150 solution; (D) 0.25 wt % SGA150–0.25 wt % alginate; and (E) 0.25 wt % MC156S–1 wt % CNC.

**Figure 3:**
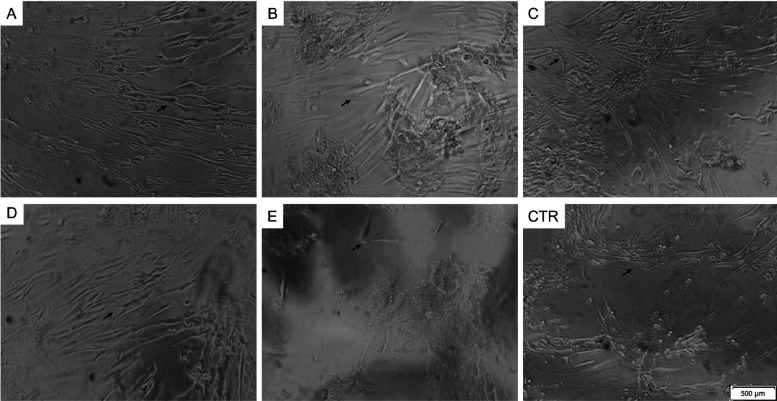
Morphology and organization of fibroblasts cultured in different cellulose hydrogels and in control conditions on culture plastic after 48 h. Black arrows point to the cells. The letters mean (CTR) control medium; (A) 0.25 wt % MC156S–0.25 wt % alginate; (B) 0.25 wt % MC156S–1 wt % CNF; (C) 0.25 wt % SGA150 solution; (D) 0.25 wt % SGA150–0.25 wt % alginate; and (E) 0.25 wt % MC156S–1 wt % CNC.

However, 0.25 wt % SGA150–0.25 wt % alginate and 0.25 wt % SGA150 pure solution at 24 and 48 h resulted in cell viability values, which were statistically lower (*p* < 0.05) than those of the control (no hydrogel) samples (Figure 2a). In the direct assay, no significant differences were observed between control and hydrogel samples at 24 and 48 h. However, 0.25 wt % MC156S–1 wt % CNF at 48 h resulted in a significant increase in cell viability compared to 0.25 wt % SGA150–0.25 wt % alginate (*p* < 0.001) and to 0.25 wt % MC156S–1 wt % CNC (*p* < 0.05). Of note, although not statistically significant (*p* > 0.05), the viability of cells cultured on 0.25 wt % SGA150–0.25 wt % alginate and 0.25 wt % MC156S–1 wt % CNC was lower than that of the control at 48 h, while that of the 0.25 wt % MC156S–1 wt % CNF was higher (Figure 2b). Interestingly, the growth in cell viability from 24 to 48 h was only statistically significant on the 0.25 wt % MC156S–0.25 wt % alginate (*p* < 0.01), 0.25 wt % MC156S–1 wt % CNF (*p* < 0.0001), and 0.25 wt % pure SGA150 (*p* < 0.01) (Figure 2c). Indeed, microscopy images showed that the cells adhered to the different hydrogels, presenting a fibroblast-like morphology similar to that of the control (Figure 3). Nevertheless, fewer cells adhered to 0.25 wt % MC156S–1 wt % CNC hydrogels (Figure 3E), corroborating the cell viability data.

### 3.2 Endotoxins Tests in Nanocellulose

**Figure 4:**
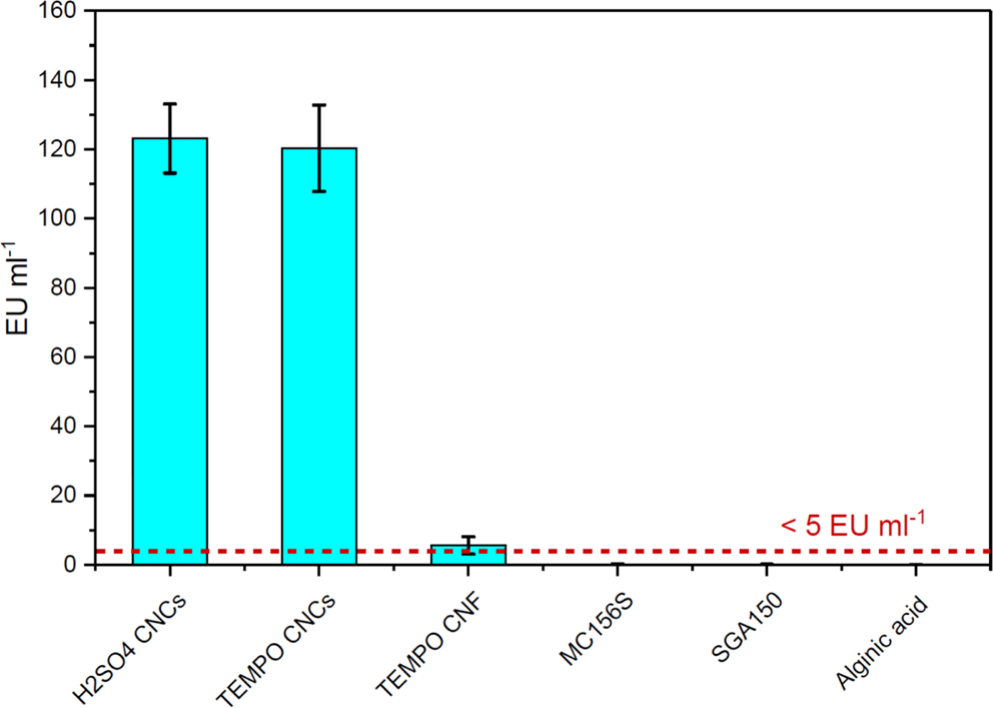
Endotoxins measured in pure solutions of H2SO_4_-hydrolyzed CNCs, TEMPO-oxidized CNC, TEMPO-oxidized CNF, methylcelluloses (MC156S, SGA150), and alginic acid dissolved in DI water.

The endotoxin levels in pure nanocellulose, methylcellulose, and alginic acid solutions were evaluated using the LAL assay after sterilization (Figure 4). From these results, the endotoxin levels in both CNC solutions were 123 ± 10 and 120 ± 12.5 EU mL^–1^, which is significantly above the limit of 5 EU mL^–1^.^28^ The observed levels of endotoxins in the CNF solution were 6 EU mL^–1^, which is slightly above the limits. The endotoxin concentrations in methylcelluloses and alginic acid were below the detectable threshold level of the assay (0.12 and 0.15 EU mL^–1^), which is considered to be endotoxin-free.

### 3.3 Mass Spectrometric Characterization of CNC and CNF Samples

The CNC and CNF samples were analyzed using negative- and positive-ion mode ESI FT-ICR MS to study the different types of polar compounds in these samples. The mass spectra of all cellulose samples are presented in the Supporting Information to this paper (Figure S1). According to these spectra, the highest peak numbers were observed for TEMPO-oxidized carboxylated CNC, ESI(−) (2914 peaks) and ESI(+) (2154) as well as sulfated CNC, ESI(−) (2,472) and ESI(+) (2391). In comparison, the peak numbers for the analyses of CNF using ESI(−)- and ESI(+)-FT-ICR-MS were slightly lower (ESI(−): 2088; ESI(+): 20,044), which might be attributable to the use of the pure CNF sample in comparison to the digested CNC samples. The mean *m*/*z* values were in a comparable range for all samples (415.34–465.87), except for the TEMPO-oxidized carboxylated CNC, ESI(+) (633.90). In this sample, between *m*/*z* 90 and 1400, multiple-charged ions are observed that are assignable, most likely, to NaCl ion clusters, which were formed during the ionization process. As a result, the formation of analyte ions is suppressed,^29,30^ resulting in a lower peak number compared to the ESI(−) data set of TEMPO-oxidized carboxylated CNC and the ESI(−)/ESI(+) data sets of sulfated CNC. The NaCl ion clusters in the TEMPO-oxidized carboxylated CNC analyzed with ESI(+), were most likely formed due to the high concentration of NaCl resulting from the digestion process.

**Figure 5:**
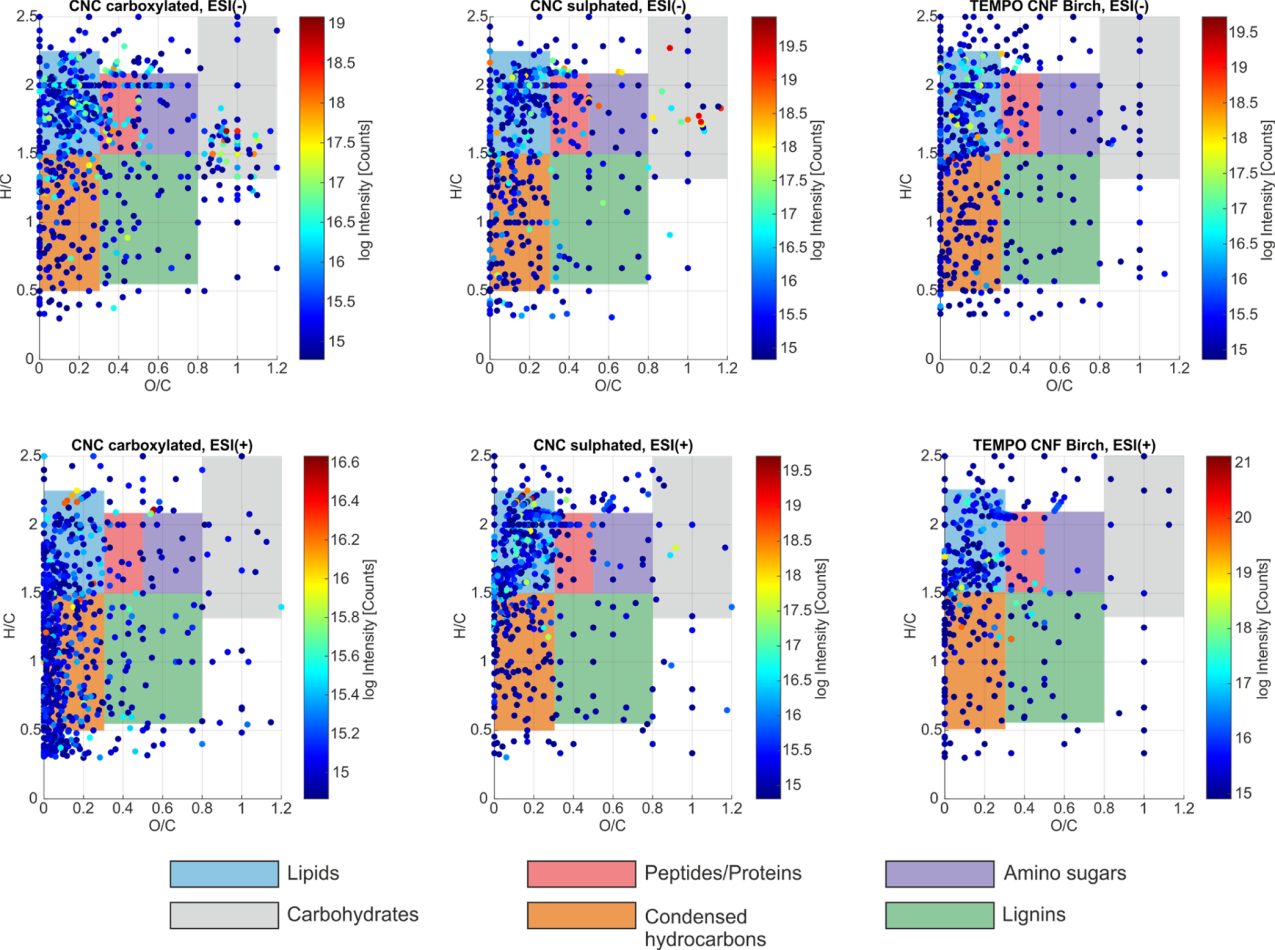
Van Krevelen plots were generated from the ESIFT-ICR-MS data of the CNC and CNF samples. The compound classes were assigned according to previous studies,^31,32^ and displayed color-coded, according to the description at the bottom. The observed intensity is presented logarithmically and color-coded (blue: low intensity, yellow: medium intensity, and red: high intensity; see the color bar).

The chemical complexity of the analyzed CNC and CNF samples was further evaluated utilizing van Krevelen plots (Figure 5). For this visualization, the O/C and H/C ratios are calculated for each assigned molecular formula. Thus, each molecule is presented as a single point in this plot. Specific O/C and H/C regions can then be correlated with different compound classes (lipids, sugars, peptides, lignins, etc.) to reveal the composition of a complex sample. In this van Krevelen plot, lignin structures can be found between O/C = 0.3–0.8 and H/C = 0.5–1.5. Condensed hydrocarbons have a similar H/C region but typically lower values in the O/C region (0–0.3). Furthermore, lipids (O/C = 0–0.3, H/C = 1.6–2.3), peptides (O/C = 0.3–0.5, H/C = 1.5–2.1), amino sugars (O/C = 0.5–0.8, H/C = 1.5–2.1), and carbohydrates (O/C = 0.8–1.3, H/C = 1.4–2.4) possess specific areas in this plot.^31,32^

According to van Krevelen plots, all analyzed samples are composed of different types of compound classes. Most of the compounds in the TEMPO-oxidized carboxylated CNC and sulfated CNC samples are assigned to the O/C and H/C regions, which correspond to lipids, peptides, amino sugars, and carbohydrates. In comparison, compounds that could be assigned to lipids and peptides/proteins are observed in the van Krevelen plots of the CNF sample.

The highest amounts of lipids and amino sugars were assigned to the data set of the TEMPO-oxidized CNC, ESI(−), whereas molecules belonging to the class of peptides were also assigned to sulfated CNC, ESI(−) and ESI(+) in an extensive amount. Cellulose-based carbohydrate molecules are assigned a significantly higher signal intensity to the TEMPO-oxidized carboxylated CNC, ESI(−) data set, compared to all other ESI FT-ICR MS analyses. The most abundant carbohydrate molecular ions in this sample mainly belong to three ion series: (a) C_12_H_18_O_12_ (*m*/*z* = 353.072550, [M-H]^−^)–C_18_H_28_O_17_ (*m*/*z* = 515.125373, [M-H]^−^); (b) C_12_H_20_O_12_ (*m*/*z* = 355.088200, [M-H]^−^)–C_18_H_30_O_17_ (*m*/*z* = 517.141023, [M-H]^−^)–C_24_H_40_O_22_ (*m*/*z* = 679.193846, [M-H]^−^); and (c) C_12_H_18_O_13_ (*m*/*z* = 369.067464, [M-H]^−^)–C_18_H_28_O_18_ (*m*/*z* = 531.120288, [M-H]^−^)–C_24_H_38_O_23_ (*m*/*z* = 693.173111, [M-H]^−^). According to these molecular formulas, mainly disaccharides and up to tetrasaccharides can be detected by ESI FT-ICR MS. In comparison to the TEMPO-oxidized carboxylated CNC, different sulfated carbohydrate molecules were detected in the sulfated CNC sample, especially using ESI(−)-MS. The most abundant ion series for this sample are (a) C_12_H_22_S_1_O_14_ (*m*/*z* = 421.065750, [M-H]^−^)–C_18_H_32_S_1_O_19_ (*m*/*z* = 583.118574, [M-H]^−^)–C_24_H_42_S_1_O_24_ (*m*/*z* = 745.171397, [M-H]^−^)–C_30_H_52_S_1_O_29_ (*m*/*z* = 907.224220, [M-H]^−^); and (b) C_15_H_26_S_1_O_16_ (*m*/*z* = 493.086879, [M-H]^−^)–C_21_H_36_S_1_O_21_ (*m*/*z* = 655.139703, [M-H]^−^)–C_27_H_46_S_1_O_26_ (*m*/*z* = 817.192526, [M-H]^−^). Hence, sulfated or sulfonated disaccharides, up to pentasaccharides, can be expected for sulfated CNCs. The potential structures for these different saccharide ion series that were detected for both the TEMPO-oxidized carboxylated and sulfated CNC are illustrated in the Supporting Information (Figure S2). Most likely, these oligosaccharides were produced from the cellulose matrix during the digestion of carboxylated and sulfated CNC because none of these molecules could be detected in the undigested CNF sample.

More in-depth information about the different compounds and compound classes, which were detected using ESI FT-ICR MS, can be obtained if these molecules are clustered into heteroatomic classes based on the assigned number of oxygen, nitrogen, and/or sulfur atoms. These heteroatomic compound clusters are then usually evaluated according to the total number of assigned molecular formulas and relative abundance of different heteroatomic classes. The visualization of these two assessment parameters for compound classes N_1_, N_2_, N_1_O_1_–N_1_O_6_, S_1_, S_2_, S_1_O_1_–S_1_O_8_, and O_1_–O_16_ is illustrated in the Supporting Information (Figure S3), as these compound classes showed the highest number of assigned molecular formulas for all analyzed samples. Nitrogen- and nitrogen-/oxygen-containing compounds (N_1_, N_2_, and N_1_O_1_–N_1_O_6_) were mainly assigned to the ESI(+) MS data sets of both TEMPO-oxidized carboxylated and sulfated CNCs, which indicate that molecules with basic functionalities but also partially to the ESI(−) MS data set of the CNF sample. In contrast, most molecular formulas in the sulfur- and sulfur-/oxygen-containing compound classes (S_1_, S_2_, and S_1_O_1_–S_1_O_8_) were assigned, unsurprisingly, to the FT-ICR-MS data sets of sulfated CNCs. The highest total number of assigned molecular formulas and relative abundances are observable for all analyzed samples in the oxygen-containing compound classes (O_1_–O_16_). Generally, the total number of these heteroatomic classes is higher for the ESI(−) MS data sets than for the ESI(+) MS data. As a result, it can be assumed that more molecules with acidic structural motifs are present in the CNC and CNF samples.

**Figure 6:**
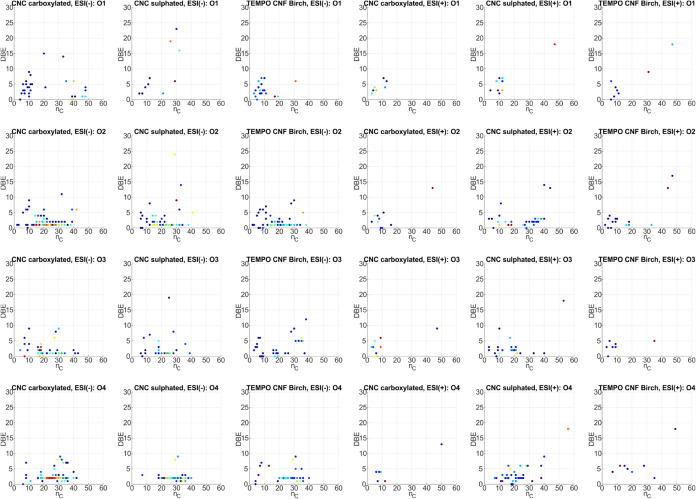
*n*_C_–DBE plots for compound classes O_1_–O_4_ of the ESI FT-ICR MS data for all three analyzed CNC and CNF samples. The observed intensity is presented logarithmically and color-coded (blue: low intensity, yellow: medium intensity, and red: high intensity).

Structural information about the different heteroatomic classes and compounds, which were clustered in these classes, was derivable by utilizing the visualization of the carbon number (*n*_C_) vs the double bond equivalent (DBE). Both molecular properties are obtained using the molecular formula assigned to an ion. The number of carbon atoms gives the *n*_C_ value, whereas DBE can be calculated according to the number of assigned carbon (*c*), hydrogen (*h*), and nitrogen atoms (*n*)
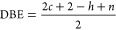
1The DBE values correspond to the number of double bonds and cycles present in the molecular structure. In contrast, the *n*_*C*_ values are used to assess the alkylation degree of a compound class.^33^ The *n*_C_–DBE plots for the most abundant heteroatomic classes, O_1_–O_4_, of the analyzed CNCs and CNF are illustrated in Figure 6. Further plots for the heteroatomic classes O_5_–O_8_, O_9_–O_12_, N_1_O_1_–N_1_O_6_, S_1_O_1_–S_1_O_4_, and S_1_O_5_–S_1_O_8_ are provided in the Supporting Information (Figures S4–S8).

Most molecules in heteroatomic classes, O_1_–O_4_ possess DBE values between 1 and 3, which are attributable to the lipid structures. For instance, molecules in the heteroatomic class O_2_ with DBE = 1, which were detected by ESI(−) FT-ICR-MS, are, most likely, saturated monocarboxylic acids. The higher DBE values (DBE ≥ 2) in this class for the ESI(−) MS data sets could correspond to unsaturated monocarboxylic acids. The saturated and unsaturated monocarboxylic acids have *n*_C_ values between 12 and 38. Additionally, in heteroatomic class O_3_, monocarboxylic acids with ether or hydroxyl groups are imaginable at DBE = 1 (*n*_C_ = 16–43) and oxo-carboxylic acids at DBE = 2 (*n*_C_ = 16–40). Dicarboxylic acids can also be expected according to the ESI(−) MS data sets in heteroatomic class O_4_ at DBE = 2 (*n*_C_ = 16–42). Besides these acidic molecules, other compounds and compound classes are assumable, especially according to the presented *n*_C_–DBE plots of heteroatomic classes O_2_–O_4_ of the ESI(+) FT-ICR-MS analysis of sulfated CNCs. For example, saturated and unsaturated ester molecules are assumable (O_2_, DBE = 1–3, *n*_C_ = 10–40), as well as molecules with multiple oxygen-containing functional groups (hydroxy, ether, aldehyde, carbonyl, ester, and carboxyl) (O_3_–O_4_, DBE = 1–4, *n*_C_ = 11–40). Thus, the lipid fraction of sulfated CNCs seems to not only contain fatty acids but also fatty esters, whereas the lipid fractions of TEMPO-oxidized carboxylated CNC and CNF are more dominated by fatty acids.^33−35^

Higher, mostly unsaturated, oxygen-functionalized molecules with DBE = 1–6 are also assumable, especially for TEMPO-oxidized and sulfated CNCs, according to the *n*_C_–DBE plots of heteroatomic classes O_5_–O_12_ (Figures S4 and S5).^33,34^ Furthermore, nitrogen-functionalized oxygen-containing compounds with DBE = 0–5 can be suggested based on the plots of heteroatomic classes N_1_O_1_–N_1_O_6_ (Figure S6). Most of these molecules were detected for sulfated CNC and CNF in classes N_1_O_1_–N_1_O_3_ using ESI(+) MS, which can be attributed to molecules with basic amino-functionalized aliphatic structures that might contain different oxygen-containing functional groups (hydroxyl, ether, aldehyde, carbonyl, ester, and carboxyl).^35−37^

Sulfur-/oxygen-containing compounds are, as expected, mostly observable in the ESI(−) and ESI(+) FT-ICR-MS data sets of sulfated CNC. Most of these compounds are part of the heteroatomic classes S_1_O_3_–S_1_O_6_ (see Figures S7 and S8). Unsaturated or aromatic sulfonic acids are assumable according to the *n*_*C*_–DBE plots of heteroatomic class S_1_O_3_ (DBE ≥ 4). In addition, sulfates and oxygen-functionalized sulfonic acids are potential structures for compounds of the heteroatomic class S_1_O_4_ at DBE = 1. DBE values of one and two in heteroatomic classes S_1_O_5_ and S_1_O_6_ are attributable to (multiply) oxygen-functionalized sulfonic acids, sulfonates, or sulfates.^38^

## 4 Discussion

In this study, human dermal fibroblasts were used as a cell model due to their key role in wound healing.^39,40^ Also, different methodologies have been used to assess cell viability. A resazurin-based assay was preferred over all other methodologies since it allows the evaluation of viability with time in the same sample without causing cell lysis using indirect and direct assay methods.^41^ The indirect assay method in this study allowed us to confirm that none of the studied hydrogels could be considered cytotoxic (reduction in cell viability of more than 30%). However, in a direct assay, where cells are seeded on the hydrogels, the same was observed for the MC156S-based hydrogel but the gels with CNC or CNF presented opposite results.

The literature recommends removing endotoxins at high temperatures (>250 °C for more than 30 min), or alkalis or acids of at least 0.1 M strength.^6,42^ Sterilization at high temperatures is less desirable for nanocellulose-based hydrogels since CNC and CNF can be easily denatured at elevated temperatures. In the present study, CNCs tend to have a high endotoxin level of more than 200 EU mL^–1^, whereas the EU standards require biobased materials to contain less than 5 EU mL^–1^.^43^ The endotoxin limits for the use of materials in the manufacturing of medical devices are between 2.15 and 20 EU pro devices, respectively, which require both new endotoxin removal methods and analytical procedures for endotoxin identification.

While the presence of CNF induced an increase in cell viability, the presence of CNC significantly reduced cell viability. In the previous literature, endotoxins and morphology of CNCs were used as explanations for the cytotoxic behavior of hydrogels.^10^ Even though the CNC size can enhance the cytotoxicity of hydrogels to cells, the impact of dimensions was excluded in this study.^23^ Both CNC samples used here had smaller lengths and mean diameters (*L:* 190 ± 50 nm; *d:* 7 ± 5 nm and *L*: 103 ± 63 nm; *d*: 15 ± 5 nm) than those in other similar studies.^44^ Although neither CNF nor CNC is considered to present relevant cytotoxicity, there have been reports of diluted CNCs being cytotoxic to murine embryo fibroblast culture depending on the size and dose.^44^ Similarly, cotton CNFs at concentrations above 200 μg/mL have been shown to reduce bovine fibroblast viability.^45^ In previous studies, the cytotoxicity of cellulose-based hydrogels was related to the presence of (1,3)-β-d-glucans.^43^ The present results indicate the presence of monocarboxylic acids, which are bound to long-chain fatty acids (C12:0 to C22:0) that might be a new environmental marker of endotoxins. The aliphatic monocarboxylic acids liberated from naturally occurring plant-based fats and oils by hydrolysis can bind to a fatty acid, forming an endotoxin. The endotoxins detected here do not contain the phosphate group as a part of the lipid-based membrane compared to previous studies.^46^ The lipid A of endotoxins could have a structure similar to that of *Rhizobium etli*, *Thiobacillus ferrooxidans*, and/or *Aquifex aeolicus*, which lack phosphate residues due to the late-functioning phosphatases on the outer surface of the inner membrane.^47,48^ Instead of phosphates, *T. ferrooxidans*, and/or *A. aeolicus* might contain galacturonic acid moieties and d-glucose residues at lipid A and core positions. The lipophilic part of endotoxins contains hydrophilic sulfates, making the entire structure more hydrophobic, and, thus, more resistant to antibiotics and sterilization.^49^ The present study demonstrates that acid hydrolysis for producing CNCs, followed by sterilization using an autoclave and UV treatment for 40 min, is not sufficient to remove the endotoxins completely. The fatty acids (e.g., lauric, myristic, palmitic, and 3-hydroxymyristic) were bound to the backbone of lipopolysaccharides through the esters, making it easier for the endotoxin structure to break into smaller fragments.^50^ The additional difficulty can be in the overall endotoxin structure that possesses hydrophobic, hydrophilic, and charged regions, by providing unique features with respect to possible interactions with other molecules. The efficient reduction of endotoxins will require the development of new techniques (e.g., high pressure and plasma treatment), which will consider the unique chemical composition of each endotoxin type without causing significant changes in the hydrogel properties (e.g., stiffness, injectability, and biocompatibility).

Previous research has identified Limulus amebocyte lysate (LAL) and rabbit pyrogen tests as suitable methods to characterize the endotoxin concentrations,^13^ whereas this study showed that FT-ICR-MS is a useful method for the identification of the endotoxin composition. FT-ICR-MS has several advantages to the other spectroscopic approaches: (a) no pretreatment of nanocellulose samples is needed; (b) no acid or base hydrolysis nor other chemical derivatization steps are necessary; (c) microgram quantities of samples are required for analyses; (d) information on other lipid A substituents, e.g., fatty esters, sulfates, sulfonates, and amino acid groups may be obtained; and (e) FT-ICR-MS is not time-consuming and is highly specific, easily analyzing both ester- and amide-linked fatty acids. In this study, both negative- and positive-ion mode ESI FT-ICR MS provided complementary information for the structural compounds of nanocellulose and methylcelluloses in the range of 200–1400 *m*/*z*. In addition, oligosaccharides, which are usually less favorably ionized in the ESI process compared to molecules with acidic or basic functionalities, could presumably be detected during our analyses for the digested carboxylated and sulfated CNC sample.^51^ The negative ionization mode provided information about chemical groups present in Lipid A, whereas l-rhamnose and galactose to which O-antigen can be attached were studied using ESI(+) FT-ICR-MS analyses.^52^ Both ionization modes utilized in this study benefited the detection of sulfonic and amino groups during FT-ICR-MS analysis of endotoxins, indicating the necessity to perform combined mode measurements.

In a previous study, bacterial nanocellulose in combination with cell-derived adhesion proteins was used as a three-dimensional scaffold in tissue engineering.^53^ Despite the high concentrations of identified endotoxins in bacterial nanocellulose, the integrated proteins activated stronger cell adhesion in three-dimensional scaffolds. In addition, the fibroblast cells that adhered to the bacterial nanocellulose surface exhibited high mitochondrial activity and high cell populations per cubic millimeter. According to the present results, a new fingerprint method was developed to identify endotoxins in cellulose-based hydrogels. Future studies will focus on exploring the role of endotoxins in biobased materials with integrated proteins, e.g., collagens and fibronectin, in cell adhesion and proliferation to biomimic the surface chemistry of the hydrogel extracellular matrix using novel combined fingerprinting methods.

## 5 Conclusions

A novel, robust, and feasible methodology was developed to biomimic the chemical surface of soft ECM and immobilize cell-derived adhesion proteins from fibroblasts. Methylcellulose, alginate, cellulose nanocrystal (CNC), and cellulose nanofiber (CNF) hydrogels were successfully fabricated using osmotic dehydration and further used as scaffolds for human dermal fibroblast cells. We observed that all hydrogels were biocompatible; nevertheless, while the presence of CNFs potentially increased cell viability, the presence of CNCs potentially reduced it. The chemical nature of endotoxins was characterized using both positive- and negative-ion mode ESI FT-ICR MS, indicating the absence of phosphate-containing groups in Lipid A. The sulfate or sulfonate groups were suggested to make the endotoxin structure more hydrophobic and resistant to sterilization. The outcomes of this study require the development of new nondestructive high-pressure methods considering the unique endotoxin composition, which will not cause significant changes in scaffold properties (e.g., stiffness, injectability, and biocompatibility).

## Supporting Information Available

Mass spectra of CNC and CNF samples with a detailed structural composition according to the ESI FT-ICR MS measurements (Figures S1–S8); mass spectra for the heteroatoms, e.g., nitrogen, oxygen, and sulfur, supporting the results on the compositional differences between carboxylated, sulfated CNCs and CNFs; *n*–DBE plots for nitrogen (N_1_O_1_–N_2_O_6_) and sulfur (S_1_O_5_–S_2_O_8_)-containing compounds (PDF)
