# Supplementary material for: Fast, Easy, and Reproducible Fingerprint Methods for Endotoxin Characterization in Nanocellulose and Alginate-Based Hydrogel Scaffolds
Source: Biomacromolecules. 2024 Sep 12;25(10):6762–72. doi: 10.1021/acs.biomac.4c00989 (PMC11480981; doi:10.1021/acs.biomac.4c00989)
Supplement: Supplementary file 1 — bm4c00989_si_001.pdf [file bm4c00989_si_001.pdf]

Supporting Information for the manuscript *Fast, Easy, and Reproducible Fingerprint Methods of Endotoxin Characterization in Nanocellulose and Alginate-based Hydrogel Scaffolds*

Figures S1-S8 illustrate the mass spectra of CNC and CNF samples with the detailed structural composition according to the ESI-FT-ICR-MS measurements. The mass spectra for the heteroatoms, e.g., nitrogen, oxygen, and sulphur are shown to support the results on the compositional differences between carboxylated, sulphated CNCs and CNF. The n-DBE plots are illustrated for nitrogen (N_1O_1-N_2O_6) and sulphur ($S_1O_5 – S_2O_8$)-containing compounds.

S1. Mass spectra of CNC and CNF samples

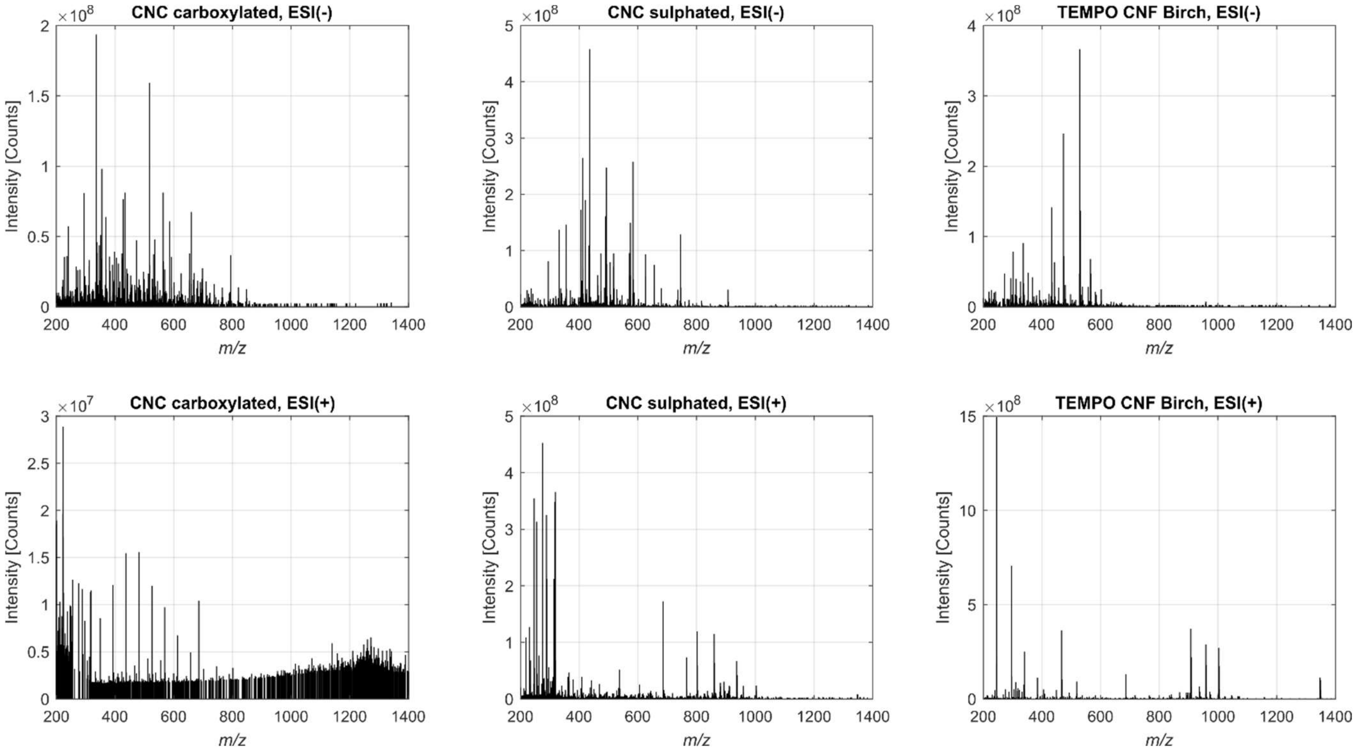

Figure S1: Mass spectra of CNF, TEMPO-oxidized and sulphated CNCs, which were analysed by ESI-FT-ICR-MS.

S2. Structural suggestions for saccharides detected in carboxylated and sulphated CNCs

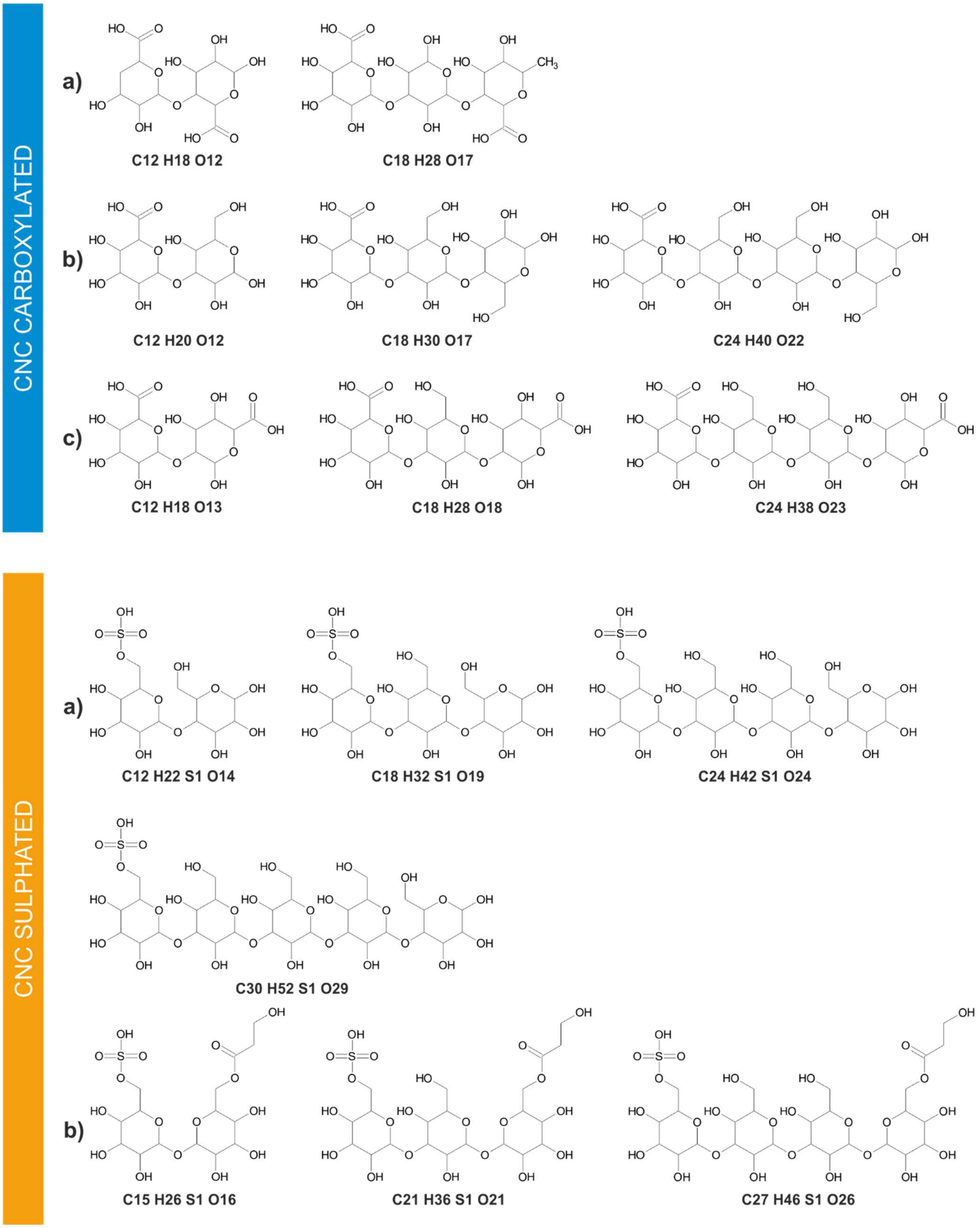

Figure S2: Structural assumptions for disaccharides up to pentasaccharides, which were detected as different ion series for TEMPO-oxidized carboxylated CNC (a – c) and sulphated CNC (a – b).

S3. Statistical comparison of heteroatomic classes

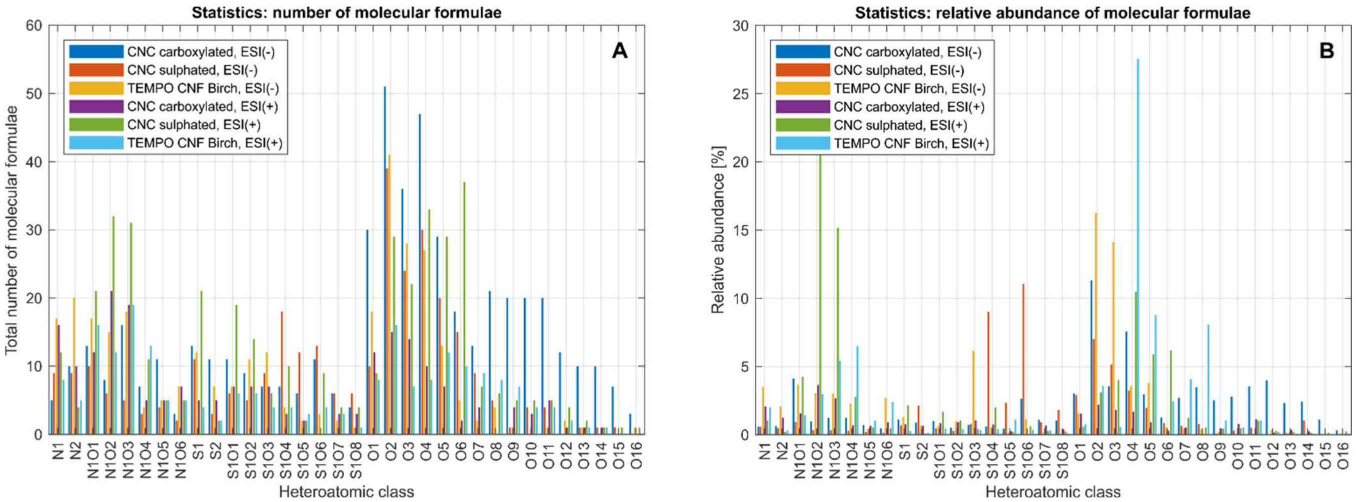

Figure S3: Comparison of assigned number of molecular formulae (A) and relative abundancies (B) to the FT-ICR-MS data sets of the CNCs and CNF for compound classes N_1, N_2, N_1O_1 – N_1O_6, S_1, S_2, S_1O_1 – S_1O_8 and O_1 – O_{16}.

S4. Further *n_C-DBE* plots for oxygen-, nitrogen-/oxygen- and sulphur-/oxygen-containing heteroatomic classes

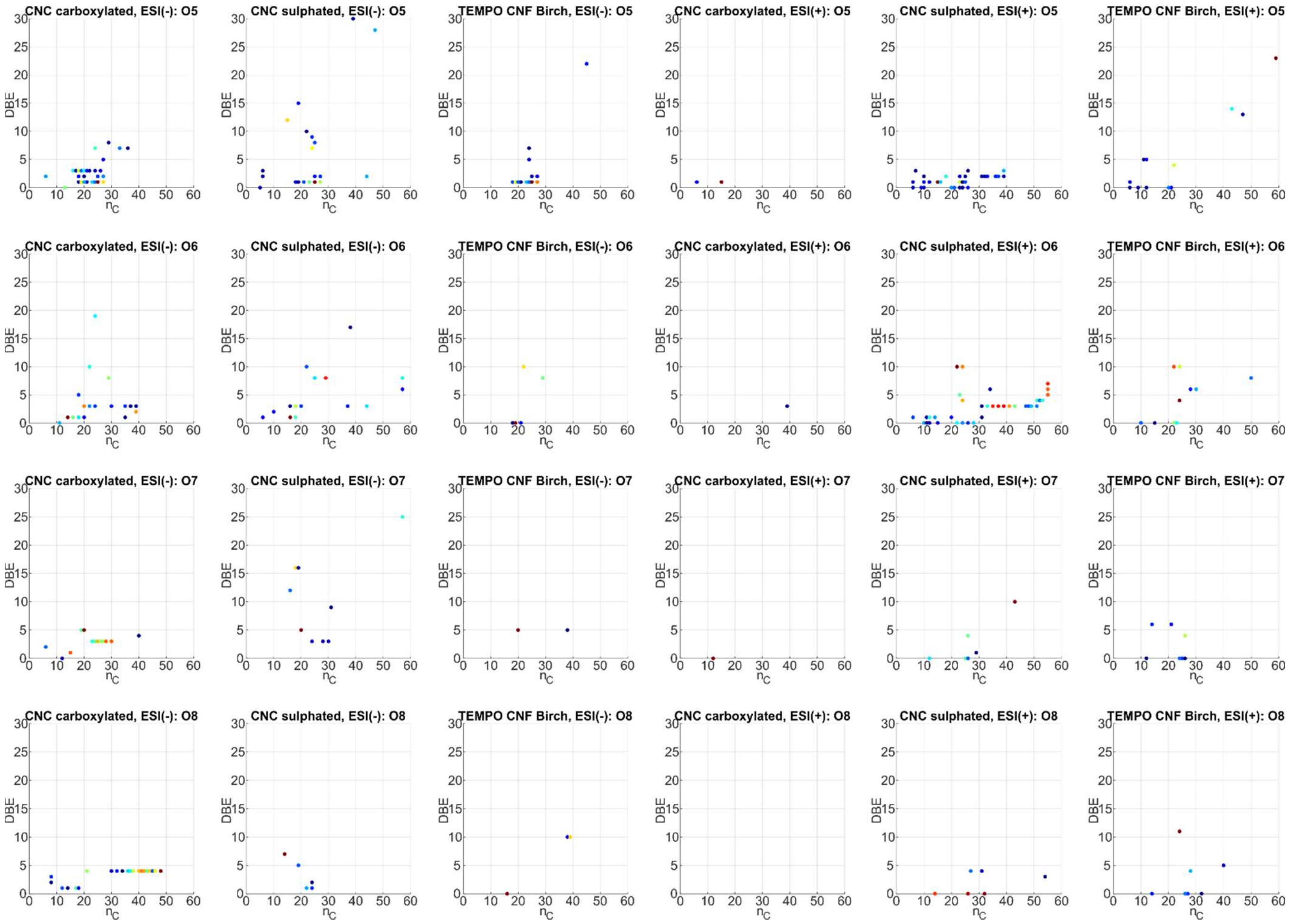

Figure S4: n_C-DBE plots for compound classes O_5 – O_8 of the ESI-FT-ICR-MS data of all three analysed CNC and CNF samples The observed intensity is presented logarithmically and colour-coded (blue: low intensity, yellow: medium intensity, red: high intensity).

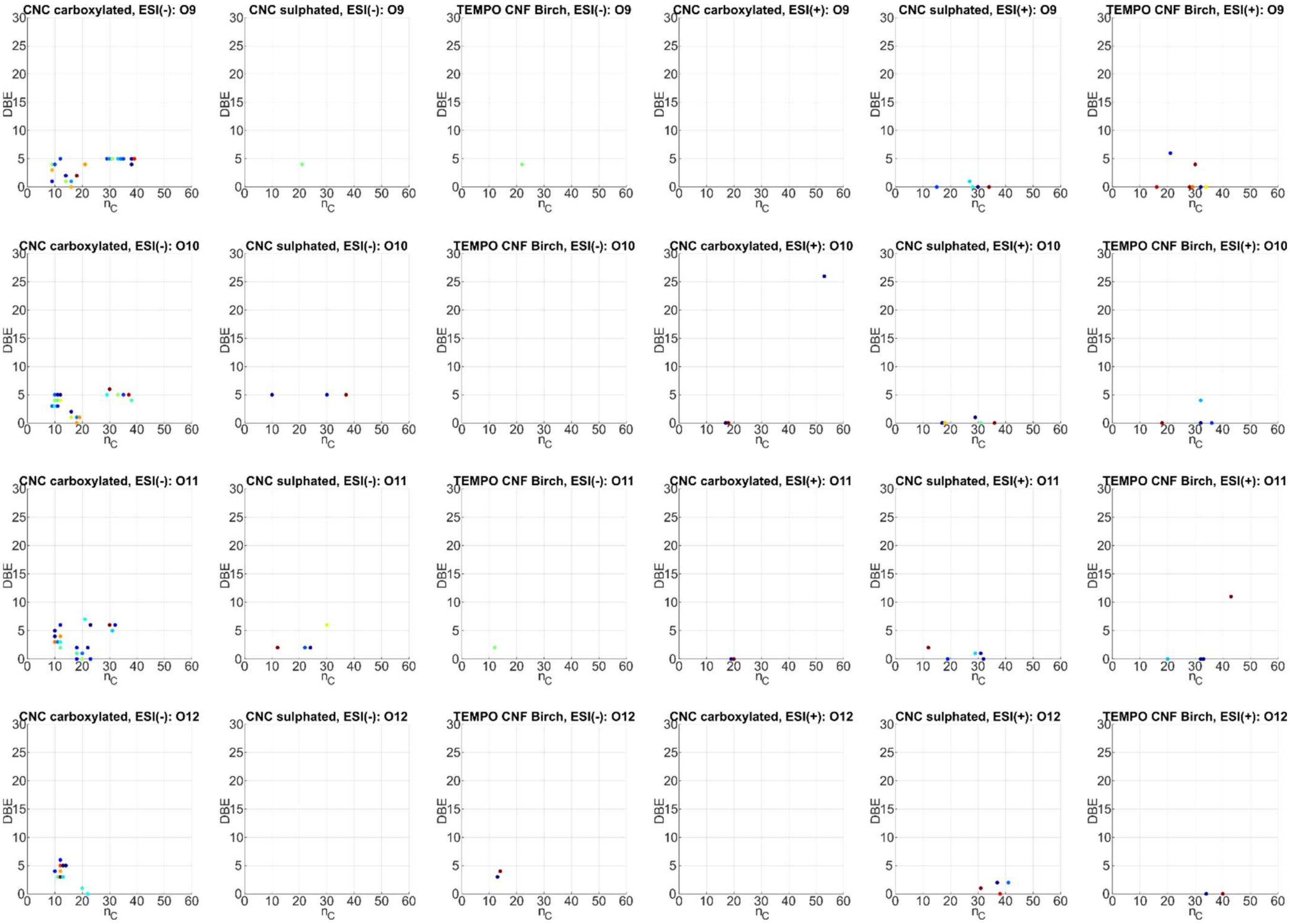

Figure S5: n_C-DBE plots for compound classes O_9 – O_{12} of the ESI-FT-ICR-MS data of CNF, TEMPO-oxidized CNC and sulphated CNC.

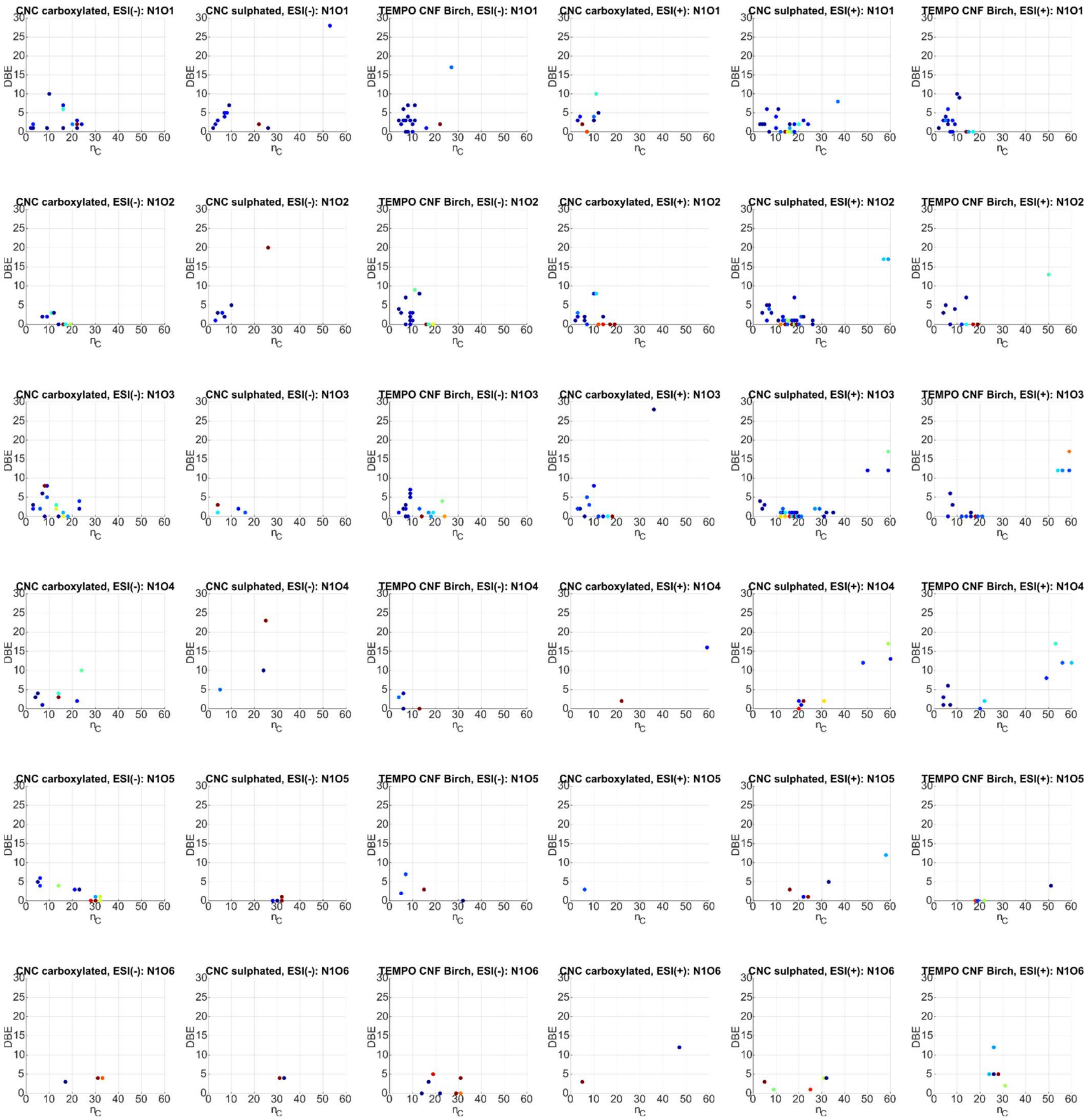

Figure S6: n_C-DBE plots for compound classes N_1O_1 – N_1O_6 of the ESI-FT-ICR-MS data of all three analysed CNC and CNF samples.

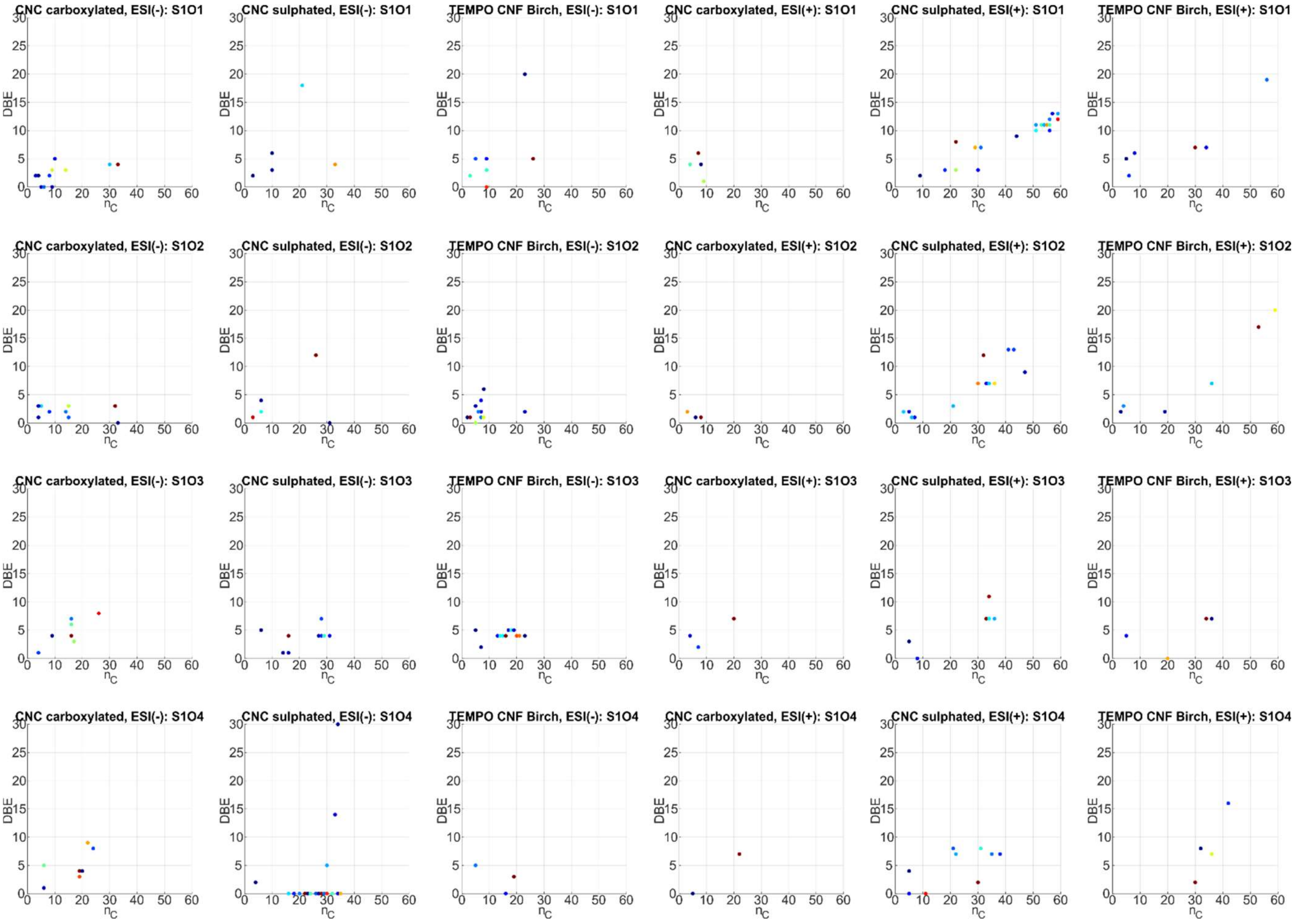

Figure S7: n_C-DBE plots for compound classes S_1O_1 – S_1O_4 of the ESI-FT-ICR-MS data of CNF, TEMPO-oxidized and sulphated CNC.

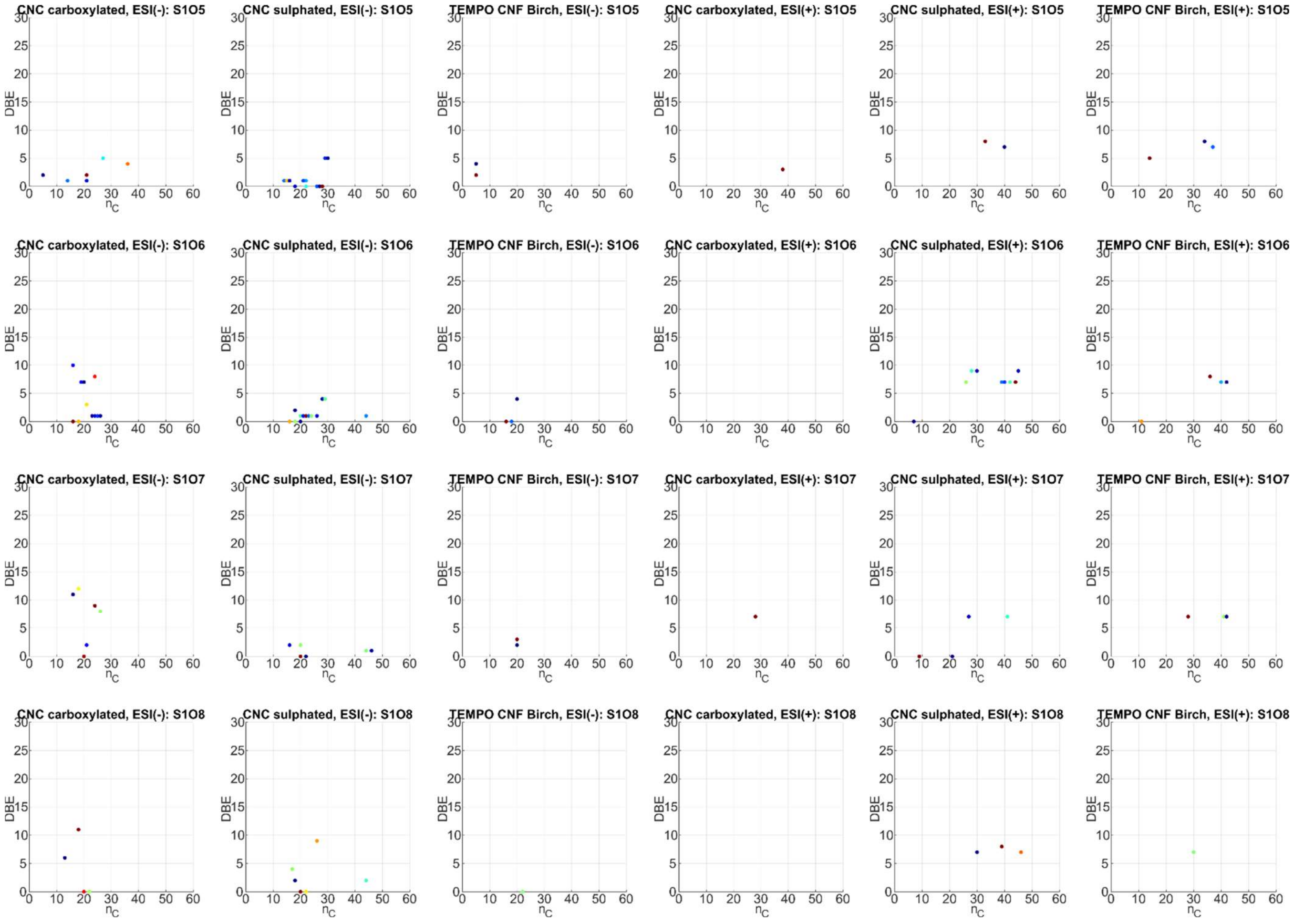

Figure S8: n_C-DBE plots for compound classes S_1O_5 – S_1O_8 of the ESI-FT-ICR-MS data of all analysed CNC and CNF samples.
